# Six-Year, Real-World Use of Prophylaxis with Recombinant Factor IX–Albumin Fusion Protein (rIX-FP) in Persons with Hemophilia B: A Single-Center Retrospective–Prospective Study

**DOI:** 10.3390/jcm13051518

**Published:** 2024-03-06

**Authors:** Antonio Coppola, Gianna Franca Rivolta, Gabriele Quintavalle, Annalisa Matichecchia, Federica Riccardi, Rossana Rossi, Anna Benegiamo, Paola Ranalli, Valeria Coluccio, Annarita Tagliaferri

**Affiliations:** 1 Regional Reference Center for Inherited Bleeding Disorders, University Hospital of Parma, 43126 Parma, Italy; grivolta@ao.pr.it (G.F.R.); gquintavalle@ao.pr.it (G.Q.); amatichecchia@ao.pr.it (A.M.); friccardi@ao.pr.it (F.R.); atagliaferri@ao.pr.it (A.T.); 2 Coagulation Laboratory, Diagnostic Department, University Hospital of Parma, 43126 Parma, Italy; rorossi@ao.pr.it (R.R.); abenegiamo@ao.pr.it (A.B.); 3 Hemophilia and Blood Rare Disease Center, Oncohematology Department, Spirito Santo Civil Hospital, 65124 Pescara, Italy; paola.ranalli@asl.pe.it; 4 Hematology Unit, University Hospital of Modena, 41124 Modena, Italy; coluccio.valeria@aou.mo.it

**Keywords:** extended half-life FIX concentrates, hemophilia B, prophylaxis, treatment outcomes, treatment personalization

## Abstract

**Background**: Extended half-life (EHL) factor IX (FIX) concentrates allow for prophylaxis with prolonged dosing intervals and high bleeding protection in persons with hemophilia B. Long-term real-world studies are lacking. **Methods**: In a retrospective–prospective study, the six-year use of prophylaxis with the EHL recombinant FIX–albumin fusion protein (rIX-FP) was analyzed, comparing outcomes with previous standard half-life (SHL) FIX in patients already on prophylaxis. **Results**: Prophylaxis with rIX-FP was prescribed in 15 patients (10 severe, 5 moderate; follow-up: 57 ± 17 months). Based on a pharmacokinetic assessment and clinical needs, the first regimen was 47 ± 7 IU/Kg every 9 ± 2 days. All but one patient remained on rIX-FP prophylaxis, adjusting infusion frequency and/or dose; the last prescribed frequency was ≥10 days in 10/13 patients, being reduced in seven and increased in four vs. the first regimen. The weekly FIX dose was unchanged; FIX trough levels were >5% in all patients. The annual infusion number and FIX IU/Kg significantly decreased (~60%) in eight patients previously on SHL FIX prophylaxis, with similar concentrate costs. Very low bleeding rates (most traumatic bleeds and the last quartile of the infusion interval), improved orthopedic and pain scores, unchanged HEAD-US scores and problem joints, and high treatment adherence (>90%) and satisfaction were registered. **Conclusions**: Personalized, carefully adjusted rIX-FP regimens contribute to the diffusion and optimization of prophylaxis in persons with severe and moderate hemophilia B, with long-term favorable bleeding, joint, and patient-reported outcomes.

## 1. Introduction

Regular prophylaxis, i.e., the long-term administration of therapeutic products aimed at preventing bleeding, particularly joint hemorrhages, with their deleterious impact on muscle–skeletal status and quality of life, is currently recommended in patients with severe hemophilia A and B (i.e., congenital deficiencies of coagulation factor [F] VIII or IX < 1%) and in moderate patients (FVIII/FIX 1–5%) with a severe bleeding phenotype [1]. Unlike hemophilia A, for which the first ‘non-replacement’ product, subcutaneously administered, is also available [2], intravenous injections of replacement factor concentrates are the mainstay of prophylaxis in hemophilia B [3]. The introduction of extended half-life (EHL) recombinant FIX (rFIX) concentrates, with relevant improvements of pharmacokinetic (PK) properties compared with standard half-life (SHL) concentrates [4], allowed for prolonged dosing intervals (up to 14–21 days vs. 2–4 days) and factor trough levels higher than those previously achieved, increasingly recognized to be inadequate to prevent all (clinical and subclinical) bleeding and the progression of arthropathy [5].

Currently, three EHL rFIX products are available in Italy, as well in many countries [4]. Two rFIX–fusion proteins, with an Fc fragment of IgG1 (rFIX-Fc, eftrenonacog alfa, Alprolix^®^, Swedish Orphan Biovitrum, Stockolm, Sweden) and with albumin (rIX-FP, albutrepenonacog alfa, Idelvion^®^, CSL Behring, Marburg, Germany), respectively, have been approved since 2017 for patients of all ages, while a glycopegylated rFIX, nonacog beta pegol (N9-GP, Refixia^®^, Novo Nordisk, Bagsvaerd, Denmark), has been more recently licensed for patients aged >12 years. Beyond molecular characteristics, these three products show considerably different PK profiles, although for all, improved PK and clinical advantages over SHL FIX have been clearly reported in phase III trials [4,6,7,8], as well in increasing real-world experience [9,10,11,12]. No head-to-head studies have been conducted and only indirect comparisons of trials or cross-over PK studies [13] are available; thus, the choice of products mainly relies on center/physician clinical experience and the interpretation of data from the literature. Moreover, available studies provide limited patient follow-up; thus, data on the long-term management and outcomes of prophylaxis with such products are still substantially lacking.

For these reasons, we aimed at analyzing data from patients on prophylaxis with rFIX–fusion protein (rIX-FP, albutrepenonacog alfa, Idelvion^®^) over more than 6 years of clinical use at our center.

## 2. Materials and Methods

### 2.1. Study Design and Subjects

In the frame of a single-center ongoing observational retrospective–prospective study on the real-world use of EHL concentrates, approved by the Area Vasta Emilia Nord (AVEN) Ethics Committee (1079/2018/OSS/AOUPR), all patients who were prescribed prophylaxis with rIX-FP at the Regional Reference Center for Inherited Bleeding Disorders, University Hospital of Parma, Italy, since the market availability (January 2017) to September 2023 and signed their informed consent were considered for this analysis. For patients already on regular prophylaxis with SHL FIX concentrates, a comparable period for the previously used product was evaluated.

Data were collected at patient enrolment (according to the study protocol, at least 6 months after starting rIX-FP treatment) retrospectively, since the product switch, and prospectively thereafter. According to the observational design, the decision to start/switch to rIX-FP prophylaxis was based on the specific clinical needs and shared with patients (or minors’ parents/guardians) after thorough discussions of benefits and possible risks. As recommended by national guidelines [14], informed consent to the use of the EHL concentrate was achieved and patients were asked to undergo a single-dose PK study to assess the individual response. A careful clinical and laboratory follow-up was planned to monitor the efficacy and safety of treatment. These practices at the center [15] are reported in the following paragraphs.

### 2.2. PK Study and Laboratory Measurements

According to expert recommendations [14,16], a PK assessment was carried out by infusing 45–50 IU/Kg of rIX-FP in less than 10 min, in non-bleeding state, after at least a 4-d wash-out. Blood samples for FIX coagulant activity (FIX:C), measured with a one-stage coagulation assay (HemosIL SynthASil, Instrumentation Laboratory/Werfen, Bedford, MA, USA), were collected at baseline and 1 and 6 h after the end of the infusion and up to 10 days thereafter, usually at 24, 48, 72, 120, 168, and 240 h. At baseline, the anti-FIX inhibitor was tested by the Bethesda method with Nijmegen modification using the above-mentioned FIX:C assay. For PK analyses, the non-compartmental model was evaluated using the Phoenix WinNonlin software package, version 8.3 (Pharsight Corp., Mountain View, CA, USA). The terminal half-life (HL), area under the curve from the time of infusion to the last measurement (AUC), clearance (normalized by body weight), volume of distribution at steady state (VSS), and mean residence time (MRT) were calculated. Incremental in vivo recovery (IVR) was obtained by dividing the maximum observed FIX:C level and the infused dose.

### 2.3. Prophylaxis Regimens and Clinical and Laboratory Follow-Up

The first rIX-FP prophylaxis regimens were prescribed based on PK assessment and the individual needs of bleeding protection (considering bleeding phenotype and risks, related to joint status and lifestyle), aimed at achieving FIX trough levels of at least >3–5%, according to the recent international recommendations [1,17]. After starting regular prophylaxis, outpatient visits and laboratory assessments were planned monthly for 3 months and every 3 months thereafter, at least up to 12 mo. of treatment. The routine 4 mo (children)/6 mo (adults) follow-up was then resumed. All visits were generally scheduled at the longest prophylaxis dosing interval to perform blood sampling for FIX trough level assessment. The mean results of the last 2 measurements for both rIX-FP (on last prescribed regimen) and of SHL FIX, where appropriate, are reported.

### 2.4. Data Collection and Outcome Assessment

All patients’ clinical data were collected through the institutional web-based clinical record xl’Emofilia^®^ (University Hospital of Parma, Parma, Italy and Arko, Magenta, Italy) [18]. Characteristics at enrolment (severity of disease, FIX:C levels, age, inhibitor history, treatment regimens, duration and type of prophylaxis) were drawn, along with historical and follow-up treatment information, including data about breakthrough bleeds (type and severity, spontaneous/post-traumatic), invasive procedures or surgeries, FIX trough levels, and FIX concentrate administrations. Indeed, beyond hospital infusions, xl’Emofilia^®^ allows patients or their caregivers to regularly register home treatment and center physicians to validate data.

Efficacy outcomes included annualized bleeding rates (ABRs), with details about joint bleeds (AJBR) and spontaneous episodes (AsBR), as well as FIX trough levels and adherence to treatment, calculated as the percentage ratio between the registered and prescribed FIX infusions. Joint status outcomes were evaluated by comparing clinical and ultrasound assessments of the six index joints (elbows, knees, and ankles) obtained at the annual multidisciplinary check-up before starting rIX-FP prophylaxis and at the last visit over the study period. The Hemophilia Joint Health Score (HJHS, version 2.1) was considered for pediatric patients; in adults in whom the HJHS was validated recently [19], for consistency with previous evaluations, the World Federation of Hemophilia Orthopedic Joint Score (or Gilbert Score) [20] was still used until the end of 2023. The Hemophilia Early Arthropathy Detection with UltraSound (HEAD-US) scores were obtained by the simplified scanning procedures developed by Martinoli et al. [21]. Joints with 3 or more bleeds in a 6-month period were defined as target joints [22], while those with chronic pain and/or a limited range of motion due to compromised integrity, irrespective of bleeding, were considered ‘problem joints’, as recently proposed [23].

The number of infusions and FIX concentrate consumption on prophylaxis were calculated for the last prescribed rIX-FP and, if appropriate, SHL concentrate regimens. FIX treatment costs were similarly evaluated, considering the current ex-factory prices of FIX concentrates in Italy.

Safety outcomes included anti-FIX inhibitor assessments and adverse events related to treatment, if any.

The treatment satisfaction of patients on rIX-FP and the previous SHL FIX prophylaxis was compared through the validated Hemo-sat questionnaire [24], routinely used at the center for monitoring changes to treatment regimens or products. The questionnaire, consisting of 34 items about six dimensions of treatment (’ease and convenience’, ‘efficacy’, ‘burden’, ‘specialist’, ‘center’, and ‘general satisfaction’), was administered before the switch and after at least 6 months of rIX-FP prophylaxis. Each-domain and global scores are provided; in all cases, the lower the score, the higher the treatment satisfaction.

### 2.5. Statistical Analysis

As descriptive statistics, numbers and percentages for categorical variables and the mean and 1 standard deviation (SD) for continuous variables are reported. The Chi-square statistics or Fisher’s Exact Test, if appropriate, were used to evaluate differences in categorical variables. Continuous variables were analyzed by the Student t test, for paired or independent samples, as appropriate. For all analyses, performed using IBM SPSS statistics (version 22), *p* values < 0.05 were considered as statistically significant.

## 3. Results

### 3.1. Patient Cohort

Over the six-year study period, regular prophylaxis with rIX-FP was prescribed in 15 patients, whose clinical characteristics and treatment regimens are reported in Table 1. The patients were all Caucasian males, aged 40.0 ± 12.6 yrs (mean ± 1 standard deviation; 2 <18 yrs), 10 with severe and 5 moderate hemophilia B, previously treated with SHL FIX concentrates (nine on long-term prophylaxis, six on demand), all but one the rFIX nonacog alfa. The remaining patient (#7) was on prophylaxis with a plasma-derived FIX concentrate purified with monoclonal antibody affinity chromatography. The mean dosing in patients on prophylaxis was 45.8 ± 9.6 IU/Kg, twice weekly in seven patients and every 3 days in two. No patient had an inhibitor history.

Table 1 also shows reasons to switch to/start rIX-FP prophylaxis and the first prescribed regimens. In all patients, the reduction in infusion burden on prophylaxis was considered for the clinical decision, being crucial for prophylaxis feasibility in those previously treated on demand; in parallel, needs for increased bleeding protection included active lifestyle/sport/work activities (n = 7), severe/evolving joint deterioration (n = 7), life-threatening bleeding episodes (n = 1), or concomitant high-risk therapy (n = 1). Additional issues were improving adherence in two patients and venous access problems in one.

### 3.2. Pharmacokinetics of rIX-FP

**Figure 1:**
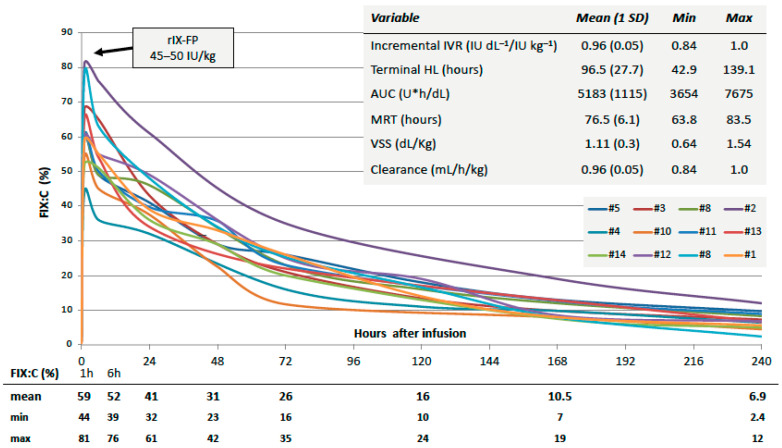
Single-dose pharmacokinetic profiles of 12 patients. Abbreviations: AUC, area under the curve; FIX:C, plasma factor IX coagulant activity (one-stage assay); HL: half-life; IVR, in vivo recovery; MRT, mean residence time; VSS, volume of distribution at steady state.

Individual PK profiles of 12 out of the 15 patients are shown in Figure 1. A complete assessment was not available in the remaining three patients.

While the IVR was consistently at about 1 IU dL^−1^/IU Kg^−1^, high but largely variable terminal HL values were found (mean of about 96 h, range of 43–139 h). The shortest values were found in the two adolescent patients (approximately 43 and 61 h). The MRT also showed high but more homogeneous values among patients. VSS was more than two-fold larger than the theoretical plasma volume, reflecting possible extravascular distribution. Overall, 5 days after the 45–50 IU/Kg rIX-FP infusion, the mean FIX:C was >15% (≥10% in all patients); after 7 and 10 days, the mean FIX:C remained at >10% and >6%, respectively; FIX:C values <5% were found only in two patients at the longest interval after infusion.

### 3.3. Prophylaxis Regimens with rIX-FP, FIX Consumption, and Cost

Based on individual PK profiles and clinical needs, the first prescribed rIX-FP prophylaxis regimens consisted of infusions of 46 ± 7 IU/Kg every 9 ± 2 days (Table 1).

Individual infusion frequencies are represented in Table 2, compared with previous SHL FIX regimens (A) and with the last prescribed regimens over the study follow-up (B).

The nine patients already on prophylaxis with SHL FIX concentrates prolonged their dosing intervals to 7–10 days on rIX-FP, while five patients previously treated on demand started rIX-FP prophylaxis with infusions every 10–14 days. Overall, 9 out of 14 patients (64%) were prescribed prophylaxis regimens with intervals ≥10 days (Table 2, A).

All but one patient remained on rIX-FP prophylaxis over the study follow-up (mean of 51 months, significantly longer in patients already on prophylaxis, ≥65 months). Indeed, an adolescent patient (#8, Table 1) discontinued treatment after 5 months, when apparently spontaneous hematomas occurred and abnormally reduced IVR was shown, in the absence of detectable anti-FIX inhibitors; the patient reverted to the previous SHL rFIX prophylaxis regimen, resuming clinical efficacy and FIX IVR. Overall, during the study period, the rIX-FP prophylaxis regimen was unchanged only in one patient; the infusion frequency was modified in 12/13 patients and dosing in 5 (in three mainly due to weight adjustments).

Considering the last prescribed rIX-FP prophylaxis regimens, the mean dosing interval was further prolonged in seven patients, up to 14 days in four of them; on the other hand, four patients had their infusion frequency increased due to breakthrough bleeds, three patients with severe arthropathy and a young patient practicing sport (Table 2, B). The latter was the only patient receiving rIX-FP at intervals <7 days (every 5 days), while 10/13 patients (77%) were on prophylaxis every ≥ 10 days.

Further treatment characteristics are summarized in Table 3, which also separately shows data from patients already on prophylaxis (n = 8), comparing rIX-FP and the previous SHL FIX product. The infusion frequency and weekly FIX dose were significantly reduced (*p* = 0.0001) on rIX-FP compared to SHL FIX prophylaxis. Further reductions in the last vs. first rIX-FP regimens were not statistically different.

Switching to/starting prophylaxis with rIX-FP (last prescribed regimen) required a mean of 37 infusions per year (range: 26–73), with an annual mean FIX consumption of 127 × 10^3^ IU/Kg (range 78–219) and costs of about EUR 226,000. These regimens resulted in a mean 61 ± 17% reduction in annual infusion burden for patients previously on SHL rFIX prophylaxis (*p* = 0.0001) and similar reductions in annual FIX IU consumption (mean −60 ± 18%; *p* = 0.0001); due to the about 2.6-fold higher rIX-FP cost per IU than SHL rFIX, a slight (not statistically significant) increase in the mean annual FIX concentrate costs for prophylaxis was calculated (about EUR 249,000 vs. 234,000; mean +6%, *p* = 0.43).

### 3.4. Outcomes of Prophylaxis with rIX-FP

The FIX trough levels were consistently higher than 5% in all patients at measurements soon after starting rIX-FP prophylaxis and showed a significant increase (*p* = 0.001) after at least 3 mo. of regular treatment (Table 3); these trough levels were remarkably higher than those reported for SHL FIX prophylaxis (*p* = 0.0005).

Adherence to the prescribed rIX-FP regimens was 87–100% in patients already on prophylaxis, among whom two had values <80% on the previous SHL FIX regimen; all patients previously treated on demand showed full adherence (100%), with the exception of a moderate patient (#13, Table 1), who needed to start prophylaxis due to a target joint and severe arthropathy; this patient often reported longer infusion intervals than those prescribed.

#### 3.4.1. Bleeding Rates and On-Demand Treatment

Over the study period, the mean ABR was low (0.7), being >1 in four patients and 0 in three patients. The mean AJBR and AsBR were even lower, being >1 in two and no patients, respectively, with higher rates of patients reporting 0 bleeds for both (Table 3). The bleeding rates further improved when only the last prescribed rIX-FP prophylaxis regimen was considered. However, the mean bleeding rates were slightly higher in patients already on regular prophylaxis compared with the SHL FIX regimens, with the differences not being statistically significant.

Overall, 36 breakthrough bleeding episodes were reported on rIX-FP prophylaxis (Table 4), the large majority after trauma, including those affecting joints. More than half of bleeds were treated with one additional rFIX-FP infusion, whereas in six and two episodes (22%), respectively, three or four infusions were given.

**Figure 2:**
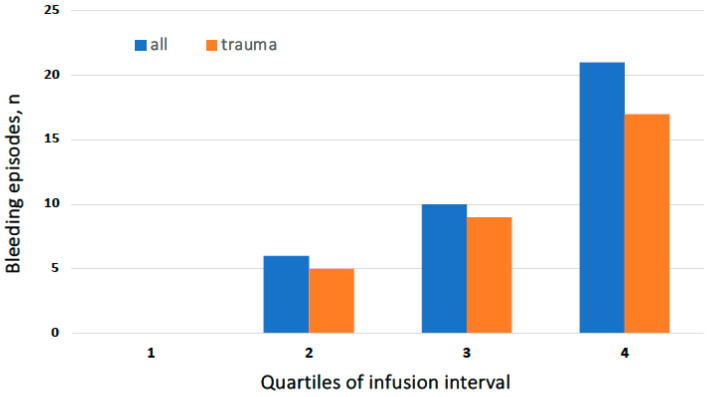
Distribution of numbers of all-type (blue columns) and traumatic (orange columns) bleeding episodes during rIX-FP prophylaxis according to the quartiles of the infusion interval. Two episodes occurring over the prescribed interval (2 and 3 days, respectively) are included in the last (4) quartile.

A direct relationship between the timing of bleeding episodes and the duration of the interval from the last prophylaxis infusion was found: the longer the elapsed interval, the higher the number of breakthrough bleeds. Indeed, approximately 60% of all bleeds (21/36) and two-thirds of apparently spontaneous events (four out of six) occurred in the last quartile of the infusion interval (Figure 2).

Dental procedures and intra-articular injections were safely performed with a single infusion (Table 4), usually on the scheduled prophylaxis day. Two minor surgeries were covered with rIX-FP doses on days 1, 3, and 6, without bleeding complications. The same patient underwent a total replacement of both hips, receiving a mean of 13 rIX-FP infusions over the first 21 post-operative days. Intra-operative bleeding and transfusion requirements were considered comparable to those in the general population by the orthopedic surgeon.

#### 3.4.2. Joint Outcomes

After the mean 51-month follow-up (Table 5) in 12 patients with at least three annual assessments, the mean physical examination scores were substantially unchanged, yet with reductions in patients previously treated on demand; the pain scores were more generally improved, with the difference reaching statistical significance and six patients scoring 0 at their last assessments (vs. three before treatment start).

**Figure 3:**
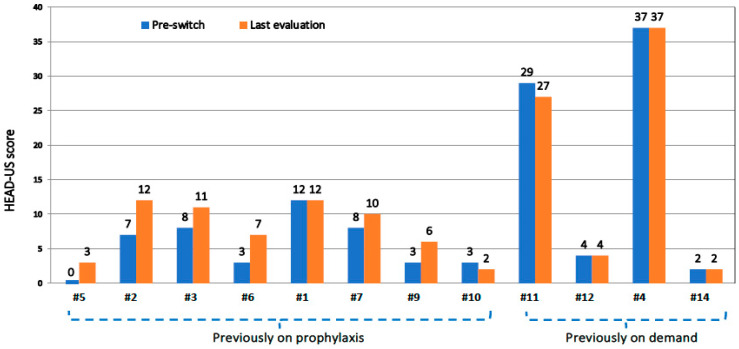
Comparison of HEAD-US scores in 12 patients over the study period, before switching to rIX-FP (blue columns) and at last assessment on rIX-FP prophylaxis (orange columns). Patients are grouped according to the previous treatment regimen (prophylaxis and on demand).

The mean HEAD-US scores were not statistically different at the pre-switch and last assessments (Table 4). As shown in Figure 3, the scores slightly increased in patients already on prophylaxis switching to rIX-FP regimens with infusion intervals >10 days (six out of eight), whereas they were stable or lower in the two patients on prophylaxis regimens with higher infusion frequencies and in all patients previously treated on demand (four out of four). Overall, the scores were stable or even improved in about three-quarters of evaluated joints (Table 5).

No target joint was reported over the study follow-up. The number of problem joints remained substantially stable, being 0 in three patients vs. one at treatment start. In parallel, the number of patients regularly practicing sport or physical activities increased (six vs. two).

#### 3.4.3. Safety

Over the study period, except for the abnormally reduced IVR mentioned above, no adverse event clearly related to rFIX-FP was reported. In that patient, as well in all enrolled patients, no inhibitor development was registered over the mean 145 exposure days (EDs, range: 36–268); eight patients reached >150 EDs while three had <50 EDs.

#### 3.4.4. Treatment Satisfaction

The Hemo-sat questionnaires, obtained from patients already on prophylaxis before switching to rIX-FP and after at least 6 months of treatment, showed that treatment satisfaction was significantly improved on rFIX-FP prophylaxis (Table 6). Indeed, both the total score and those from the four domains mainly reflecting the impact of pharmacological therapy (ease and convenience, efficacy, burden, general satisfaction) were significantly lower on rIX-FP prophylaxis than on the previous SHL FIX regimen. The highest improvement was reported in the ‘burden’ domain.

## 4. Discussion

The introduction of EHL factor concentrates positively impacted the management of prophylaxis in persons with hemophilia, especially those with hemophilia B [1,3]. Indeed, EHL rFIX concentrates showed a greater improvement of PK profiles than EHL rFVIII, with a three-to-five-fold extension of half-life and reduction in clearance compared with SHL products, thus allowing for a relevant reduction in the treatment burden of intravenous injections and, in parallel, an increase in FIX trough levels [1,4].

Real-world studies are confirming the clinical benefits shown in pivotal trials in terms of reduced infusion frequency and high protection from bleeding in patients on prophylaxis with EHL rFIX concentrates [9,10,11,12]. However, beyond the limitations due to the rarity of hemophilia B, clinical choices cannot rely on rigorous comparisons among the available products, and data on the long-term management and outcomes of prophylaxis with such concentrates are still substantially lacking. Sharing and updating real-world experience is therefore crucial for increasing knowledge and optimizing clinical practice. In this perspective, we decided to analyze data from our patients on prophylaxis with rIX-FP; due to the longer market availability and the center’s experience, this patient group was the largest on EHL rFIX prophylaxis in our ongoing retrospective–prospective study investigating the efficacy and safety of EHL products. Moreover, a considerable six-year period of clinical use, adopting a careful clinical and laboratory patient follow-up [15], was available.

A meaningful finding from our analysis is the actual diffusion of prophylaxis in patients with hemophilia B, especially those with moderate disease, in agreement with current national and international guidelines [1,14]. In our cohort, rFIX-FP prophylaxis became feasible in six patients (40%) previously reluctant or unable due to the high treatment burden with SHL FIX. All but one patient showed very high adherence to treatment and achieved bleeding and joint protection, even greater than patients already on prophylaxis. These findings are consistent with data on tertiary/late secondary prophylaxis, showing clinical benefits of these regimens, although started in adolescent/adult patients [1,25], and the awareness of clinical improvements in those patients long treated on demand.

In the whole cohort, rIX-FP enabled the personalization of prophylaxis, especially through the flexibility of infusion frequency, able to address both the individual needs of bleeding and joint protection and convenience issues, including venous access and adherence problems [1,26,27,28]. Our data provide further real-world evidence of the efficacy of rIX-FP prophylaxis regimens with a dosing frequency from 5 to 14 days, in heterogeneous clinical conditions, including severe joint deterioration, high bleeding risk due to strenuous physical activity or sport, and even concomitant anticoagulation/antiplatelet treatment. Higher protection is achieved by the overall improved PK profiles, including high FIX trough levels, as also documented in our patients. These levels are often well above the currently recommended 3–5% [1,17], and show significant increases after ≥3 months of regular treatment (means of 8.3% then 9.8%). Consistent with the PK data in the literature, in our patients, mean FIX levels at an infusion frequency of 5 days could be maintained above 15%. By analyzing the bleeding phenotype of patients with mild and moderate hemophilia A, above this level, no joint bleeding should be expected [29]. However, optimal trough levels on prophylaxis are largely debated and thought to be variable according to the patients’ bleeding phenotype, levels of physical activity, and joint status [1,5,30]. As an example, in the lack of evidence, experts consider that trough levels from about 5% to almost 50% are needed for the safe practice of sports, depending on the activity risk category and the absence or presence of joint damage [31]. This issue is now frequently addressed in clinical practice in patients with preserved/improved joint status on prophylaxis and a growing awareness of benefits of physical activity and sport. The number of patients regularly practicing physical activity increased over the study period even in our cohort.

Reduced bleeding rates and higher proportions of patients with 0 bleeds are often reported in patients on prophylaxis with rIX-FP than on the previous SHL FIX regimen [6,9,11,12]; this was not observed in our patients, who were on highly effective pre-switch prophylaxis (mean ABR: 0.4) and had a longer follow-up. Although not statistically significant, a slight increase in breakthrough bleeds was reported after switching to rIX-FP; most bleeding episodes occurred after trauma and in the last quartile of the infusion interval. The safety of rIX-FP regimens up to 21 days has been documented [28]; however, despite improved trough levels and AUC, the prolonged lack of peak levels on regimens with a low infusion frequency may result in bleeding risks, especially in patients with an active lifestyle or joint damage. For this reason, although most patients maintained or even prolonged their infusion intervals over this study, in four patients, the infusion frequency was increased, with improved bleeding protection. The optimization of prophylaxis regimens [1,15,25] should confront reductions in treatment burden, recognized as the key feature determining patients’/caregivers’ treatment preference, more important than efficacy [32,33].

The need for a careful clinical follow-up is further highlighted by the trend of increasing HEAD-US scores in our study, in particular in patients already on prophylaxis who switched to low-frequency rIX-FP regimens. Although not statistically significant, these findings may reflect an early/evolving joint damage, in which subclinical bleeding may play a role [1]. However, our long-term observation provides data on stable orthopedic joint scores and even a reduced impact of joint morbidity, with improved pain scores and an unchanged number of problem joints [23]. The current minimization of bleeding by prophylaxis regimens, as shown in our cohort, aims to extend the assessment of treatment outcomes and value beyond the ABR and target joints [34]. Along this line, early signs/markers of joint damage, as well of more reliable functional and patient-reported outcomes (including treatment satisfaction, as reported in our study) should be identified, also considering innovative products recently introduced in or approaching clinical practice in hemophilia, i.e., non-replacement agents and gene therapy [2,3].

In our 6-year clinical use of rIX-FP, as expected in previously treated patients, no inhibitor development was reported, including in the adolescent who showed reduced clinical efficacy and FIX IVR 5 months after switch, which then normalized resuming the previous SHL rFIX concentrate. These phenomena were likely related to anti-drug antibodies (ADAs), which rarely exert neutralizing effects [35]. Laboratory tests for ADAs are not routinely available; therefore, we were not able to further characterize such neutralizing interferences. Again, this unusual adverse event emphasizes the importance of close monitoring in patients switching to new therapeutic products.

The relatively small patient population is a major limitation of this study; however, the accuracy and reliability of long-term, mainly prospective, outcome data and the homogeneous clinical practice strengthen our findings.

In conclusion, this study provides clear real-world evidence that personalized, carefully adjusted rIX-FP regimens significantly contribute to the diffusion and optimization of prophylaxis in persons with severe and moderate hemophilia B. The relevant reduction in treatment burden is associated with long-term adherence, bleeding protection, and favorable joint and patient-reported outcomes.

## Figures and Tables

**Table 1:** Patient’s clinical characteristics, regimens of SHL rFIX prophylaxis, and reasons to switch to rIX-FP with first prescribed regimen.

Patient	FIX:C (%)	Age (yrs)	Treatment Regimen before rIX-FP Start ^†^	Age at Start of Prophy (yrs)	Prophylaxis Duration (yrs)	SHL FIX Regimen		Reason(s) ^§^ to Switch	First rIX-FP Regimen		Follow-Up (mo.)
						Dose (IU/kg)	Frequency		Dose (IU/kg)	Frequency	
1	<1	53	Tertiary prophy	30	23	44	2 per week	Protection ^¶^	43	every 7 d	76
2	<1	40	Tertiary prophy	17	23	55	2 per week	Protection *	54	every 7 d	75
3	<1	42	Tertiary prophy	18	24	50	every 3 d	Protection ^¶^, adherence	47	every 10 d	74
4	<1	65	On demand	59	6	NA	NA	Prophylaxis feasibility ^¶,††^	43	every 10 d	73
5	<1	29	Secondary prophy	6	23	27	2 per week	Protection *	32 ^	every 10 d	73
6	2	30	Secondary prophy	11	19	35	every 3 d	Protection *	48	every 10 d	65
7	<1	45	Tertiary prophy	33	12	50	2 per week	Protection ^¶^	50	every 10 d	27
8	<1	13	Primary prophy	2	11	44	2 per week	Venous access, protection	54	every 7 d	5
9	<1	34	Tertiary prophy	16	18	50	2 per week	Protection *	47	every 7 d	75
10	<1	16	Primary prophy	2	14	57	2 per week	Protection *, venous access	45	every 7 d	58
11	2.5	42	On demand	38	4.5	NA	NA	Prophylaxis feasibility ^¶,§§^	50	every 10 d	57
12	<1	55	On demand	53	2.5	NA	NA	Prophylaxis feasibility *^,¶¶^	45	every 14 d	33
13	1.1	36	On demand	33	3	NA	NA	Protection ^¶^, adherence	47	every 10 d	30
14	1.6	16	On demand	14	2	NA	NA	Protection ^¶,‡^	52	every 14 d	31
15	1.2	76	On demand	75	1	NA	NA	Protection ^‡‡^	55	every 5 d	12
Mean		40.0		27.1	12.9	45.8	3.4		46	9.1	51
1 SD		12.6		11.2	3.9	9.6	0.2		7	2.2	25

Abbreviations: d, days; NA, not applicable; rIX-FP, recombinant FIX albumin fusion protein; SD: standard deviation; SHL: standard half-life. ^†^ According to the definitions by the World Federation of Hemophilia [1]. ^§^ In order of clinical relevance, in parallel with reduced infusion burden. ^¶^ Need for higher protection from bleeding due to severe/evolving arthropathy. * Bleeding risks due to active lifestyle, sport, or work activities. ^††^ This patient had a history of 3 intracranial bleeding episodes, but he was unable/reluctant to start prophylaxis because of difficult venous access and home treatment. ^ The patient, who lived abroad for 2 years, was switched to rIX-FP at another center. ^§§^ Beyond arthropathy, this moderate patient had a risk of falls and a need for intensive physiotherapy due to neurologic comorbidity. ^¶¶^ This severe patient with a mild bleeding phenotype had to start prophylaxis due to bleeding risk in work activities. ^‡^ Increased physical activity and detection of early signs of joint damage at ultrasound. ^‡‡^ This patient received FIX prophylaxis due to concomitant oral anticoagulants and then aspirin.

**Table 2:** Changes in infusion intervals from SHL rFIX concentrate regimens to the first prescribed rIX-FP prophylaxis regimen (A) and variations of the latter over the follow-up (B).

A. SHL FIX Regimen (Patients, n)	rIX-FP First Regimen (n = 14) *			B. rIX-FP First Regimen (Patients, n)	rIX-FP Last Regimen (n = 13) *^,¶^				
	Every 7 d	Every 10 d	Every 14 d		Every 5 d	Every 7 d	Every 10 d	Every 12 d	Every 14 d
2 per wk (n = 7)	5	2		Every 7 d (n = 4 ^¶^)	1		2		1
Every 3 d (n = 2)		2		Every 10 d (n = 7)		2	1 ^†^	1	3
On demand (n = 5)		3	2	Every 14 d (n = 2)			1 ^§^		1

Abbreviations: d, days; rIX-FP, recombinant FIX albumin fusion protein; SHL, standard half-life; wk, week. In panel B, light grey cells show patients who did not change their infusion frequency over the study follow-up; dark grey cells highlight patients who reduced the interval of prophylaxis dosing. * Patient #15 was not included because his prophylaxis regimens were adjusted according to the target through FIX levels of a different antithrombotic treatment (first with infusions every 5 days on oral anticoagulant, then every 14 days when receiving aspirin). ^¶^ Patient #8, who was prescribed rIX-FP every 7 days and discontinued treatment after 5 months due to recurrent hematomas and poor in vivo recovery, was not included. ^†^ In this patient, the prophylaxis regimen with infusions every 10 days was resumed, after receiving infusions every 12 days for more than 3 years, due to a traumatic bleeding episode and bleeding risk in work activities. ^§^ In this patient, the prophylaxis regimen was intensified, but his adherence was unsatisfactory.

**Table 3:** Treatment characteristics, FIX trough levels, bleeding rates, FIX consumption, and costs in patients on prophylaxis with rIX-FP and comparison with SHL FIX regimens in those previously on prophylaxis.

Variable ^†^		All Patients (n = 14) *	Patients Previously on Prophylaxis (n = 8)		*p*
			rIX-FP	SHL ^§^	
Treatment duration, mo.		57 ± 17	65 ± 17	62 ± 19	0.56
Exposure days		145 ± 77	192 ± 61	534 ± 95	0.0005
Weekly FIX dose, IU/Kg	first regimen	36 ± 10	40 ± 9	96 ± 30	0.001
	last regimen	34 ± 14	38 ± 15		
Infusion interval, days	first regimen	9.5 ± 2.9	8.5 ± 2.9	3.4 ± 0.2	0.001
	last regimen	10.8 ± 3.3	9.9 ± 3.4		
FIX trough, %	first month	8.3 ± 1.5	8.8 ±1.5	3.9 ± 1.3	0.0005
	>3 mo.	9.8 ± 2.0 ^	10.9 ± 1.7		
ABR, n		0.7 ± 0.7	0.9 ± 0.7	0.4 ± 0.4	0.12
AJBR, n		0.4 ± 0.5	0.5 ± 0.6	0.2 ± 0.3	0.13
AsBR, n		0.2 ± 0.2	0.2 ± 0.2	0.1 ± 0.2	0.15
Patients with ABR = 0, n (%)		3 (21)	1 (13)	3 (38)	
Patients with AJBR = 0, n (%)		5 (36)	3 (38)	6 (75)	
Patients with AsBR = 0, n (%)		9 (64)	5 (63)	7 (88)	
Infusions ^‡^, n/year		37 ± 15	42 ± 16	109 ± 8	0.0001
Adherence, %		91 ± 4	94 ± 4	89 ± 9	0.51
FIX concentrate consumption ^‡^, ×10^3^ IU/year		127 ± 38	140 ± 39	339 ± 88	0.0001
FIX concentrate costs ^‡^, ×10^3^ EUR/year		226 ± 67	234 ± 60	249 ± 70	0.43

Abbreviations: ABR, annualized bleeding rate; AJBR, annualized joint bleeding rate; AsBR, annualized spontaneous bleeding rate; rIX-FP, recombinant FIX albumin fusion protein; SHL, standard half-life concentrate. ^†^ Means ± 1 standard deviation. * Patient #15 is not included because his prophylaxis regimen was adjusted according to the specific clinical needs of hemostatic coverage of different antithrombotic treatments (oral anticoagulant, then aspirin). ^§^ Comparable time period to that of rIX-FP prophylaxis. ^‡^ Calculated according to the last prescribed rIX-FP regimen. ^ *p* = 0.001 vs. FIX trough at first month.

**Table 4:** Bleeding episodes and invasive procedures/surgeries over the study period.

Variable	
Total breakthrough bleeds, n (patients)	36 (11)
Traumatic, n (%)	30 (83)
Joint/traumatic, n (%)	20 (56)/15 (75)
Muscle hematoma, n (%)	10 (28)
Other type, n (%)	6 (16)
FIX IU consumption per bleed, mean (1 SD)	5314 (2883)
Infusions per bleed, mean (SD)	1.6 (1.0)
Bleeds treated with a single infusion, n (%)	21 (58)
Invasive procedures *, n (patients)	15 (7)
Treated with a single infusion, n (%)	15 (100)
FIX IU consumption, mean (SD)	3500 (707)
Minor surgeries ^§^, n (patients)	2 (2)
FIX IU consumption, mean	12,000
Days of treatment	3
Major surgeries ^†^, n (patients)	2 (1)
FIX IU consumption ^‡^, mean	38,500
Infusions ^†^, mean	13

* Dental procedures (n = 7, 47%) and intra-articular injections (n = 8, 53%). ^§^ Skin lesion excision and hand surgery. ^†^ Two total hip replacements in the same patient. ^‡^ Over the first 3 weeks after surgery.

**Table 5:** Joint outcomes and related variables over the study period (n = 12).

Variable ^†^	Treatment Start	Last Assessment *	*p*
Physical examination score ^§^	11.1 ± 10.8	8.0 ± 15.3	0.25
Pain score ^§^	1.8 ± 2.5	0.8 ± 1.8	0.01
HEAD-US score	10.3 ± 12.2	11.1 ± 10.8	0.08
score unchanged, n of joints (%)	44 (64)		
score increased, n of joints (%)	18 (26)		
score reduced, n of joints (%)	7 (10)		
Target joints, n (patients)	0 (0)	0 (0)	
Problem joints, n (patients)	26 (11)	24 (9)	
Sport/high-risk activities, patients	2	6	

HEAD-US, Hemophilia Early Arthropathy Detection with UltraSound. ^†^ Means ± 1 standard deviation, unless otherwise indicated. * Assessments at the last available annual check-up; mean follow-up: 51 ± 16 months. ^§^ From WFH (Gilbert) Orthopedic Joint Score in adults and HJHS 2.1 in the 2 adolescent patients.

**Table 6:** Satisfaction with treatment evaluated by the Hemo-sat questionnaire in patients on rIX-FP prophylaxis and comparison with previous prophylaxis with SHL FIX concentrates.

Hemo-Sat Dimension	Mean Score (1 SD) n = 8		*p*
	rIX-FP	SHL	
Ease and convenience	8.8 (2.8)	23.8 (12.4)	0.03
Efficacy	8.9 (11.1)	27.1 (14.7)	0.01
Burden	3.7 (3.8)	21.8 (10.6)	0.01
Specialist/nurse	5.7 (10.7)	7.9 (13.2)	0.22
Centre/hospital	3.7 (9.4)	5.5 (10.6)	0.14
General satisfaction	6.3 (8.9)	23.2 (13.9)	0.03
Total score	6.7 (4.5)	23.9 (19.2)	0.02

Abbreviations: rIX-FP, recombinant FIX albumin fusion protein; SD, standard deviation; SHL, standard half-life.

## Data Availability

The data supporting the findings from this study are available from the corresponding author upon reasonable request, in compliance with current data protection regulations.

## References

[1] Srivastava A., Santagostino E., Dougall A., Kitchen S., Sutherland M., Pipe S.W., Caraco M., Mahlangu J., Ragni M.V., Wyndiga J. (2020). World Federation of Hemophilia Guidelines for the management of hemophilia. Haemophilia.

[2] Nogami K., Shima M. (2023). Current and future therapies for haemophilia-Beyond factor replacement therapies. Br. J. Haematol..

[3] Sidonio R.F., Malec L. (2021). Hemophilia B (Factor IX deficiency). Hematol. Oncol. Clin. N. Am..

[4] Mahlangu J. (2018). Updates in clinical trial data of extended half-life recombinant factor IX products for the treatment of haemophilia B. Ther. Adv. Hematol..

[5] Oldenburg J. (2015). Optimal treatment strategies for hemophilia: Achievements and limitations of current prophylactic regimens. Blood.

[6] Santagostino E., Martinowitz U., Lissitchkov T., Pan-Petesch B., Hanabusa H., Oldenburg J., Boggio L., Negrier C., Pabinger I., von Depka Prondzinski M. (2016). Long-acting recombinant coagulation factor IX albumin fusion protein (rIX-FP) in hemophilia B: Results of a phase 3 trial. Blood.

[7] Powell J.S., Pasi K.J., Ragni M.V., Ozelo M.C., Valentino L.A., Mahlangu J.N., Josephson N.C., Perry D., Manco-Johnson M.J., Apte S. (2013). Phase 3 study of recombinant factor IX Fc fusion protein in hemophilia B. N. Engl. J. Med..

[8] Collins P.W., Young G., Knobe K., Karim F.A., Angchaisuksiri P., Banner C., Gürsel T., Mahlangu J., Matsushita T., Mauser-Bunschoten E.P. (2014). Recombinant long-acting glycopegylated factor IX in hemophilia B: A multinational randomized phase III trial. Blood.

[9] Hermans C., Marino R., Lambert C., Mangles S., Sommerer P., Rives V., Maro G., Malcangi G. (2020). Real-world utilization and bleed rates in patients with haemophilia B who switched to recombinant factor IX fusion protein (rIX-FP): A retrospective international analysis. Adv. Ther..

[10] O’Donovan M., Bergin C., Quinn E., Singleton E., Roche S., Benson J., Bird R., Byrne M., Duggan C., Gilmore R. (2021). Real-world outcomes with recombinant factor IX Fc fusion protein (rFIXFc) prophylaxis: Longitudinal follow-up in a national adult cohort. Haemophilia.

[11] Matino D., Iorio A., Keepanasseril A., Germini F., Caillaud A., Carcao M., Hews-Girard J., Iserman E., James P., Lee A. (2022). Switching to nonacog beta pegol in hemophilia B: Outcomes from a Canadian real-world, multicenter, retrospective study. Res. Pract. Thromb. Haemost..

[12] Tagliaferri A., Molinari A.C., Peyvandi F., Coppola A., Demartis F., Biasoli C., Borchiellini A., Cultrera D., De Cristofaro R., Daniele F. (2023). IDEAL study: A real-world assessment of pattern of use and clinical outcomes with recombinant factor IX albumin fusion protein (rIX-FP) in patients with haemophilia B. Haemophilia.

[13] Escuriola Ettingshausen C., Hegemann I., Simpson M.L., Cuker A., Kulkarni R., Pruthi R.K., Garly M.L., Meldgaard R.M., Persson P., Klamroth R. (2019). Favorable pharmacokinetics in hemophilia B for nonacog beta pegol versus recombinant factor IX-Fc fusion protein: A randomized trial. Res. Pract. Thromb. Hemost..

[14] Italian Association of Haemophilia Centres (AICE) (2018). Principi di Trattamento e Aggiornamento Delle Raccomandazioni per la Terapia Sostitutiva Dell’emofilia A e B. https://aiceonline.org/?p=9792.

[15] Tagliaferri A., Matichecchia A., Rivolta G.F., Riccardi F., Quintavalle G., Benegiamo A., Rossi R., Coppola A. (2020). Optimising prophylaxis outcomes and costs in haemophilia patients switching to recombinant FVIII-Fc: A single-centre real-world experience. Blood Transfus..

[16] Iorio A., Edginton A.N., Blanchette V., Blatny J., Boban A., Cnossen M., Collins P., Croteau S.E., Fischer K., Hart D.P. (2018). Performing and interpreting individual pharmacokinetic profiles in patients with hemophilia A or B: Rationale and general considerations. Res. Pract. Thromb. Haemost..

[17] Peyvandi F., Berger K., Seitz R., Hilger A., Hecquet M.-L., Wierer M., Buchheit K.-H., O’Mahony B., Bok A., Makris M. (2020). Kreuth V initiative: European consensus proposals for treatment of hemophilia using standard products, extended half-life coagulation factor concentrates and non-replacement therapies. Haematologica.

[18] Pattacini C., Rivolta G.F., Di Perna C., Riccardi A., Tagliaferri A., on behalf of Haemophilia Centres Network of Emilia-Romagna Region (2009). A web-based clinical record ‘xl’Emofilia^®^’ for outpatients with haemophilia and allied disorders in the Region of Emilia-Romagna: Features and pilot use. Haemophilia.

[19] St-Louis J., Abad A., Funk S., Tilak M., Classey S., Zourikian N., McLaughlin P., Lobet S., Hernandez G., Akins S. (2022). The Hemophilia Joint health Score version 2.1 validation in adult patients study: A multicenter international study. Res. Pract. Thromb. Haemost..

[20] Gilbert M.S. (1993). Prophylaxis: Musculoskeletal evaluation. Semin. Hematol..

[21] Martinoli C., Della Casa Alberighi O., Di Minno G., Graziano E., Molinari A.C., Pasta G., Russo G., Santagostino E., Tagliaferri A., Tagliafico A. (2013). Development of a simplified scanning procedure and scoring method for Hemophilia Early Arthropathy Detection with Ultrasound (HEAD-US). Thromb. Haemost..

[22] Blanchette V.S., Key N.S., Ljung L.R., Manco-Johnson M.J., van den Berg H.M., Srivastava A. (2014). Definitions in hemophilia: Communication from the SSC of the ISTH. J. Thromb. Haemost..

[23] Burke T., Rodriguez-Santana I., Chowdary P., Curtis R., Khair K., Laffan M., Mclaughlin P., Noone D., O’Mahony B., Pasi J. (2023). Humanistic burden of problem joints for children and adults with haemophilia. Haemophilia.

[24] Riva S., Bullinger M., Amann E., von Mackensen M. (2010). Content comparison of haemophilia specific patient-rated outcome measures with the international classification of functioning, disability and health (ICF, ICF-CY). Health Qual. Life Outcomes.

[25] Tagliaferri A., Feola G., Molinari A.C., Santoro C., Rivolta G.F., Cultrera D.B., Gagliano F., Zanon E., Mancuso M.E., Valdrè L. (2015). Benefits of prophylaxis versus on-demand treatment in adolescents and adults with severe haemophilia A: The POTTER study. Thromb. Haemost..

[26] Lambert T., Benson G., Dolan G., Hermans C., Jiménez-Yuste V., Ljung R., Morfini M., Zupančić-Šalek S., Santagostino E. (2018). Practical aspects of extended half-life products for treatment of haemophilia. Ther. Adv. Hematol..

[27] Gill J.C., Roberts J., Li Y., Castaman G. (2019). Sustained high trough factor IX activity levels with continued use of rIX-FP in adult and paediatric patients with haemophilia B. Haemophilia.

[28] Mancuso M.E., Lubetsky A., Pan-Petesch B., Lissitchkov T., Nagao A., Seifert W., Li Y., Santagostino E. (2020). Long-term safety and efficacy of rIX-FP prophylaxis with extended dosing intervals up to 21 days in adults/adolescents with hemophilia B. J. Thromb. Haemost..

[29] den Uijl I.E., Fischer K., van der Bom J.G., Grobeee D.E., Rosendal F.R., Plug I. (2011). Analysis of low frequency bleeding data: The association of joint bleeds according to baseline FVIII activity levels. Haemophilia.

[30] Iorio A., Iserman E., Blanchette V., Dolan G., Escuriola Ettingshausen C., Hermans C., Negrier C., Oldenburg J., Reininger A., Rodriguez-Merchan C. (2017). Target plasma factor levels for personalized treatment in haemophilia: A Delphi consensus statement. Haemophilia.

[31] Martin A.P., Burke T., Asghar S., Noone D., Pedra G., O’Hara J. (2020). Understanding minimum and ideal factor levels for participation in physical activities by people with haemophilia: An expert elicitation exercise. Haemophilia.

[32] Furlan R., Krishnan S., Vietri J. (2015). Patient and parent preferences for characteristics of prophylactic treatment. Patient Pref. Adh..

[33] von Mackensen S., Kalnins W., Krucker J., Weiss J., Miesbach W., Albisetti M., Pabinger I., Oldenburg J. (2017). Haemophilia patients’ unmet needs and their expectations of the new extended half-life factor concentrates. Haemophilia.

[34] Manco-Johnson M.J., Warren B.B., Buckner T.W., Funk S.M., Wang M. (2021). Outcome measures in haemophilia: Beyond ABR (Annualized Bleeding Rate). Haemophilia.

[35] Prezotti A.N.L., Frade-Guanaes J.O., Yamaguti-Hayakawa G.G., Ozelo M.C. (2022). Immunogenicity of current and new therapies for hemophilia A. Pharmaceuticals.

